# Pyrrolyl and Indolyl α-γ-Diketo Acid Derivatives Acting as Selective Inhibitors of Human Carbonic Anhydrases IX and XII

**DOI:** 10.3390/ph16020188

**Published:** 2023-01-27

**Authors:** Davide Ialongo, Antonella Messore, Valentina Noemi Madia, Valeria Tudino, Alessio Nocentini, Paola Gratteri, Simone Giovannuzzi, Claudiu T. Supuran, Alice Nicolai, Susanna Scarpa, Samanta Taurone, Michele Camarda, Marco Artico, Veronica Papa, Francesco Saccoliti, Luigi Scipione, Roberto Di Santo, Roberta Costi

**Affiliations:** 1 Istituto Pasteur-Fondazione Cenci Bolognetti, Dipartimento di Chimica e Tecnologie del Farmaco, Sapienza University of Rome, p.le Aldo Moro 5, I-00185 Rome, Italy; 2 Laboratory of Molecular Modeling Cheminformatics & QSAR, NEUROFARBA Department, University of Florence, Via U. Schiff 6, Sesto Fiorentino, 50019 Firenze, Italy; 3 NEUROFARBA Department, Pharmaceutical and Nutraceutical Section, University of Florence, Via U. Schiff 6, Sesto Fiorentino, 50019 Firenze, Italy; 4 Dipartimento Medicina Sperimentale, Sapienza University of Rome, Viale Regina Elena 324, 00161 Rome, Italy; 5 Department of Movement, Human and Health Sciences, Division of Health Sciences, University of Rome “Foro Italico”, 00135 Rome, Italy; 6 Department of Sensory Organs, Sapienza University of Rome, Viale del Policlinico 155, 00161 Rome, Italy; 7 Department of Motor Sciences and Wellness, University of Naples “Partenope”, 80133 Naples, Italy; 8 D3 PharmaChemistry, Italian Institute of Technology, Via Morego 30, 16163 Genova, Italy

**Keywords:** metalloenzyme, carbonic anhydrase, diketo acids, carbonic anhydrase inhibitors, breast cancer, tongue squamous cell carcinoma, pharynx squamous cell carcinoma, colon carcinoma

## Abstract

Solid tumors are active tissues containing hypoxic regions and producing metabolic acids. By decreasing pH, cancer cells create a hostile environment for surrounding host cells and foster tumor growth and progression. By governing acid/base regulation, carbonic anhydrases (CAs) are involved in several physiological/pathological processes, including tumors. Indeed, CAs are clinically relevant in cancer therapy as among the fifteen human isoforms, two of them, namely CA IX (overexpressed in solid tumors and associated with increased metastasis and poor prognosis) and CA XII (overexpressed in some tumors) are involved in tumorigenesis. Targeting these two isoforms is considered as a pertinent approach to develop new cancer therapeutics. Several CA inhibitors (CAIs) have been described, even though they are unselective inhibitors of different isoforms. Thus, efforts are needed to find new selective CAIs. In this work, we described new diketo acid derivatives as CAIs, with the best acting compounds **1c** and **5** as nanomolar inhibitors of CA IX and XII, being also two orders of magnitude selective over CAs I and II. Molecular modeling studies showed the different binding poses of the best acting CAIs within CA II and IX, highlighting the key structural features that could confer the ability to establish specific interactions within the enzymes. In different tumor cell lines overexpressing CA IX and XII, the tested compounds showed antiproliferative activity already at 24 h treatment, with no effects on somatic not transformed cells.

## 1. Introduction

Carbonic anhydrases (CAs) are widespread metalloenzymes present in prokaryotes and eukaryotes [1]. In mammals, at least 15 different CA isoforms have been described, with different catalytic activity, cellular compartment location and tissue distribution [2,3]. Isoforms I, II, III, VII and XIII are present in the cytosol, VA and VB in mitochondria and VI in saliva and milk. Differently, isoforms IV and XV are anchored to the plasma membranes, while IX, XII and XIV are transmembrane proteins with extracellular active sites [4,5,6]. This metalloproteins family uses Zn^2+^ as a cofactor to convert carbon dioxide to bicarbonate ions and protons. Indeed, in mammalian CAs, the active site contains a Zn^2+^ coordinated by three histidine residues and a water molecule/hydroxide ion that acts as a potent nucleophile [7].

CAs are involved in several physiological/pathological processes, including gluconeogenesis, tumorigenicity and the growth and virulence of various pathogens among others [8,9,10,11,12,13,14,15,16]. Isoforms IX and XII are localized mainly on hypoxic tumor cells [17,18,19,20], and they are involved in tumorigenesis by regulating pH inside and outside the cancer cell [21]. As a consequence, they interfere with phosphorylation processes and play a role in cell–cell adhesion [21,22]. Indeed, CA IX and XII are overexpressed in several types of carcinomas such as gliomas/ependymomas, mesotheliomas, papillary/follicular carcinomas, as well as carcinomas of the bladder, uterine cervix, kidneys, esophagus, lungs, head and neck, breast, brain, vulva and squamous/basal cell carcinomas, among others [23,24,25,26,27,28,29,30,31,32]. For instance, CA IX expression is upregulated in breast cancer, and it is considered a prognostic marker for the triple negative breast cancer phenotype [33,34]. Moreover, CA IX overexpression is significantly associated with advanced progression and poor survival in squamous cell carcinoma [35] and CA XII expression correlates with patients’ survival in colorectal carcinomas, indicating poorer prognosis [36]. Therefore, they provide a target for cancer therapy because of their specificity towards the tumor cells.

Several CA inhibitors (CAIs) have been described so far, with the sulfonamides and their isosteres (sulfamates, sulfamides, etc.), that have been used in clinical practice for more than 60 years, being the most investigated chemical classes of inhibitors [11,37]. In addition, compounds belonging to the coumarin family [37], some polyamines (such as spermine and its derivatives) [38] as well as phenols/carboxylic acid derivatives [37,39] were discovered as CAIs. However, these CAIs unselectively inhibit many of the 15 human (h)CA isoforms. Thus, efforts have to be made to find new CAIs able to selectively inhibit each isoform.

**Figure 1:**
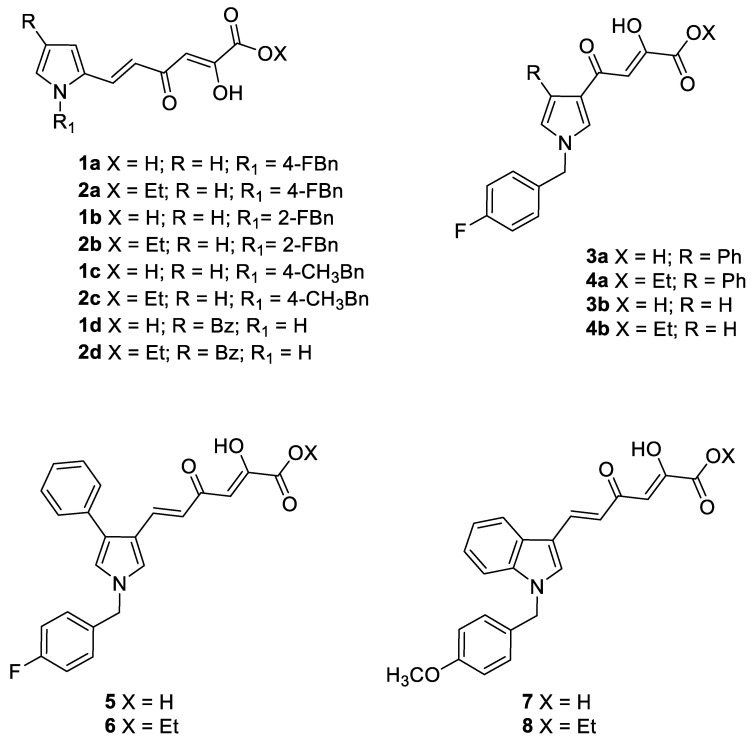
Structures of pyrrolyl and indolyl DKA derivatives **1a–d, 2a–d, 3a,b, 4a,b** and **5–8**.

Metalloenzymes, such as CA, play pivotal roles in several biological processes, making them central for the progression of many diseases, and as such, making metalloenzymes attractive targets for therapeutic intervention [40,41,42]. The vast majority of small molecules targeting metalloenzymes are reported to act via coordination of the inhibitor to the catalytic active site metals [43,44]. Among them, diketo acid derivatives (DKAs) are known for their capability of chelating metal divalent ions such as magnesium [45] or zinc ions [46]. Given our longstanding experience in the design and synthesis of DKA derivatives, we screened our in-house library of DKAs previously synthesized as divalent metal ions chelators [47]. The CA inhibition profile of compounds **1a–d, 2a–d, 3a,b, 4a,b** and **5–8** (Figure 1) was evaluated by applying a stopped flow CO_2_ hydrase assay [48] against four physiologically significant hCA isoforms (I, II, IX, and XII), in comparison with acetazolamide (AAZ) as a reference drug.

## 2. Results and Discussion

### 2.1. Chemistry

The synthesis of pyrrolyl and indolyl DKA derivatives **1a–d, 2a–d, 3 a,b, 4a,b** and **5–8** is reported in the literature [49,50,51].

### 2.2. In Vitro Enzymatic Assay

Compounds **1a–d, 2a–d, 3a,b, 4a,b** and **5–8** were tested against hCA isoforms I, II, IX and XII using a stopped flow technique. In detail, these were a series of (i) pyrrolyl derivatives characterized by the presence of a diketohexenoic chain in the 2- or 3-position of the pyrrole core (**1a–d**, **2a–d** and **5,6**), (ii) 3-diketobutanoic pyrrolyl compounds (**3a,b** and **4a,b**) and (iii) 3-diketohexenoic indoles (**7,8**). The K*_i_* values for each compound are reported in Table 1.

In general, the tested compounds proved to be selective inhibitors of the isoforms IX and XII, with respect to I and II. Indeed, nine derivatives were completely inactive against isoform I, seven against isoform II, while only one compound reported a K*_i_* of >100 µM against isoform IX and no inactive compounds were found against isoform XII out of the sixteen tested compounds. It is also worthy to note that the acid derivatives reported higher inhibitory potencies than the corresponding ester counterparts against all the tested isoforms. In detail, while in general all the acids proved to be active against the assayed CA isoforms (with the sole exception of acids **3a** and **5** that were inactive vs. CA I), all the ester compounds were inactive against CA I and II. Only one ester reported a K*_i_* lower than 40 µM against CA IX, while no ester derivative showed a K*_i_* value lower than 71 µM vs. CA XII.

Regarding isoforms I and II, we can state that better results, even though with weak potencies, were obtained in inhibiting CA II in comparison to I. The tested compounds, in fact, reported inhibitory activities in the range of 14–78 µM vs. CA II and in the range of 51–92 µM vs. CA I. The best inhibitor of CA I was the 3-diketobutanoic acid **3b**, while the best results in inhibiting CA II were obtained for 2-diketohexenoic acids **1a** and **1b**.

In general, comparable potencies were observed in inhibiting both CA IX and XII. Indeed, the tested compounds reported inhibitory activities in the range of 0.3–91 µM vs. CA IX and in the range of 0.2–92 µM vs. CA XII. Among them, 6 compounds were active in the nanomolar range (of which 2 were against CA IX and 4 against CA XII), 11 derivatives active with 1 µM < K*_i_* < 40 µM (7 against CA IX and 4 against CA XII) and 12 compounds showing K*_i_* values between 40 and 100 µM. In detail, the acid endowed with the lowest activity against both CA IX and XII was **3b** (K*_i_* values of 18.4 and 7.9 µM, respectively), while the lowest active ester derivative against CA IX was **4b** (K*_i_* > 100 µM) and **2c** against CA XII (K*_i_* = 90.8 µM). However, it is worthy to note that the acid derivatives reported a 10- to 100-fold gain in activity with respect to the ester counterparts. Indeed, the best acting compounds were the acids **1c**, **3a**, **5** and **7**, behaving as dual CA IX/XII inhibitors (**1c**, K*_i_* values of 0.8 and 0.9 µM; **3a**, K*_i_* values of 1.4 and 0.3 µM; **5**, K*_i_* values of 0.3 and 0.2 µM; **7**, K*_i_* values of 1.1 and 0.8 µM, respectively). In particular, compounds **1c** and **5** were the most potent derivatives among all the tested series of compounds, being also two orders of magnitude selective over hCAs I and II. It is also interesting to highlight that these compounds showed a better selective inhibition profile on CA IX and XII over I and II, among the tested derivatives. Noteworthy, compound **5**, despite being less active than the reference drug acetazolamide (AAZ), proved to be more selective for hCA IX vs. hCA I and II. Indeed, compound **5** was from two to three folds of magnitude more active on hCA IX than hCA II and I, respectively, while AAZ inhibited hCA II and I in the same order of magnitude or ten times less than hCA IX, respectively. The same behavior was observed for compound **5** in inhibiting hCA XII, while AAZ was from one to two folds of magnitude more active on hCA XII than hCA I and II, respectively.

By comparing the K*_i_* values of 3-diketohexenoic acid **5** with that of its diketobutanoic analogue **3a**, it is possible to observe that a gain in potency is achieved, showing compound **5** having an increase in activity by more than 4 times against CA IX and about 1.5 times against CA XII (CA IX: **5**, K*_i_* = 0.3 µM; **3a**, K*_i_* = 1.4 µM; CA XII: **5**, K*_i_* = 0.2 µM; **3a**, K*_i_* = 0.3 µM). A similar gain in activity was also observed by shifting from the indole scaffold to the 4-phenyl substituted pyrrole core as shown by the four-fold increase in the inhibitory potency of pyrrole derivative **5**, with respect to the indole **7** (CA IX: **5**, K*_i_* = 0.3 µM; **7**, K*_i_* = 1.1 µM; CA XII: **5**, K*_i_* = 0.2 µM; **7**, K*_i_* = 0.8 µM).

Within the diketobutanoic series, it is possible to observe that the introduction of a 4-phenyl ring on the pyrrolyl core led to an improvement in potency vs. both CA IX and XII, as reported by the pyrrolyl diketobutanoic derivative **3b** with respect to its 4-phenyl substituted counterpart **3a** (CA IX: **3b**, K*_i_* = 18.4 µM; **3a**, K*_i_* = 1.4 µM; CA XII: **3b**, K*_i_* = 7.9 µM; **3a**, K*_i_* = 0.3 µM).

Within the 2-diketohexenoic pyrrolyl series, it is possible to notice that the activity varies depending on the *N*-benzyl substituent, increasing in the following order: 2-F (**1b**) < 4-F (**1a**) < 4-CH_3_ (**1c**).

### 2.3. Molecular Modeling

**Figure 2:**
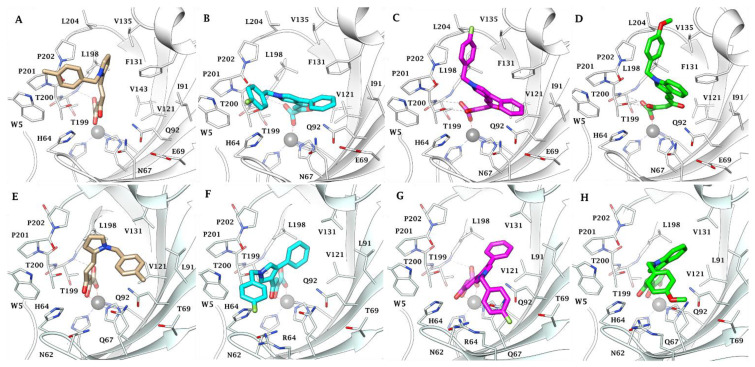
Predicted binding mode of pyrrolyl and indolyl α-γ-diketo acid derivatives **1c**, **3a**, **5** and **7** to CA II and CA IX. Active site view of CA II (white ribbon) in adduct with **1c** (**A**), **3a** (**B**), **5** (**C**) and **7** (**D**). Active site view of CA IX (light blue ribbon) in adduct with **1c** (**E**), **3a** (**F**), **5** (**G**) and **7** (**H**). The zinc(II) ion is represented as a grey sphere bound to the protein ligands H94, H96 and H119. H-bonds are represented as black dashed lines.

The binding mode of the pyrrolyl and indolyl α-γ-diketo acid derivatives was predicted in silico by docking compounds **1c**, **3a**, **5**, **7** into the active sites of the hCA IX and hCA II, as the main target and off-target isoforms, respectively. Docking studies followed by a refinement in a VSGB solvent model show that all compounds are able to occupy the catalytic region of the binding pockets by coordinating the zinc ion and interacting with the residues nearby (Figure 2). The different molecular architecture of the two active sites addresses diverse ligand binding mode, zinc coordination pattern and, as a result, significantly different inhibition profiles (Table 1). The hCA II/IX F131/V131 mutation makes the hCA IX binding site roomier than hCA II, promoting a better fitting of the ligands into the tumor-associated isozyme binding pocket. In detail, in hCA II, the carboxylate group of **1c**, **3a**, **5**, **7** coordinates the zinc ion by a tetrahedral geometry, acts as a H-bond acceptor in the binding with the backbone NH group of T199 (compounds **1c** and **3a**) and T200 (compounds **5** and **7**). The 2-oxo group of ligands **5** and **7** is additionally in H-bond contact with the side chain OH of T200. The pyrrole ring of **1c** lodges in the cleft lined by L198, P202 and F131, whereas the ligand benzyl tail forms hydrophobic contacts with P201 and P202 at the outer rim of the cavity. The shorter carboxylate-bearing chain of compound **3a** compared to **1c**, **5** and **7** prevents the ligand folding and leads its N-benzyl and 4-phenyl moieties to protrude outwards, establishing a few interactions with W5 and I91, respectively. In contrast, its diketohexenoic analogue **5** was predicted to fold in the hCA II active site. This makes the ligand N-benzyl pendant able to occupy the cleft lined by L198, P202, F131 and L204, whilst the 4-phenyl group accommodates between V121, Q92 and I91. An almost overlapping binding mode was computed for derivative **7**, with the pyrrole-fused benzene ring being in π–π contacts with F131.

The hCA II/IX F131/V131 swap reduces the steric hindrance within the binding cavity, reducing the constraints of the ligand zinc binding moiety and enabling a metal dichelation by the compound’s carboxylate and 2-oxo groups. The carboxylate function of the four ligands additionally receives an H-bond, with the side chain OH of T199 reinforcing the binding efficacy. Furthermore, in the hCA IX binding site, all ligands show lipophilic molecular portions accommodated and π-alkyl sandwiched within the cleft formed by L198, V131, V121, W209, markedly strengthening the binding: i.e., the N-benzylpyrrolyl pendant of **1c**, the 4-phenyl substituent of **3a** and **5** and the indole ring of derivative **7**. In contrast, the N-benzyl tails of **3a**, **5** and **7** were predicted to lie over the enzymatic rim formed by H64, N61 and R64. The in silico prediction supports the measured hCA IX selective inhibition profiles over hCA II. In fact, the hCA IX active site amino acid composition and architecture allow the ligands to inhibit the target isozyme more effectively than hCA II as a result of (i) a dual zinc coordination vs. a monocoordination and (ii) a wider network of VdW and hydrophobic contacts that the ligands form with the protein as a result of an improved fitting.

### 2.4. In Vitro Proliferation Assay

The effects of selected compounds were also examined on the proliferation of four cell lines from different human solid tumors overexpressing hCA isoforms IX and XII: triple negative breast cancer MDA-MB231, colon carcinoma HT-29, tongue squamous carcinoma SCC-15 and pharynx squamous carcinoma FaDu. The cell lines were treated with 5, 10, 20, 50, 100 µM of each single compound for 24 and 48 h. In parallel, the proliferation assay was performed also on HaCaT, a human non-transformed keratinocyte cell line, with each compound used at the same concentrations and times already indicated. The median values from three different proliferation assays for each compound and for each cell line were calculated and their significance was evaluated.

Compounds **1b**, **1c**, **5** and **7**, showing the best in vitro inhibition activities, were analyzed. Compound **8** was also chosen in order to compare its effects in a cellular model with that of its acidic counterpart **7**. Despite its lower enzymatic inhibition data, the ester compound **8** proved to be active in an in vitro proliferation assay in the same order of magnitude as the acid counterpart **7**. These results might be due to the higher cell membrane permeability of ester compounds compared to their acidic counterparts.

All the compounds with high hCA inhibitory activity induced significant inhibition of the proliferation to a different extent in all four tumor cell lines. In detail, compounds **7** and **8** showed the most important antiproliferative activity among the five compounds tested, by determining a significant decrease in alive cells at the lowest concentration of 10 µM already at 24 h. Compounds **1c** and **5** also showed significant antiproliferative activity, even though it was less impressive than the two previous molecules. On the other hand, compound **1b**, characterized by the lowest hCA inhibitory activity among the tested acid derivatives, exhibited a low efficacy in inhibiting the proliferation of the analyzed cell lines, exclusively at higher concentrations and times of treatment.

The half maximal effective concentration (EC_50_) for each compound and each tumor cell line at 24 and 48 h was calculated and reported in Table 2.

**Figure 3:**
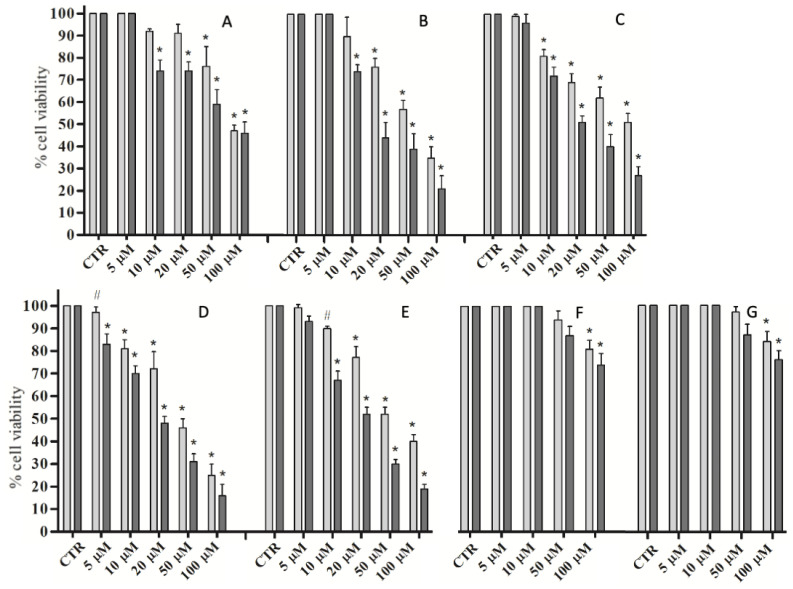
Proliferation assay of Fadu (**A**–**E**) and HaCaT (**F**,**G**) cells untreated (CTR) and treated with compound **1b** (**A**), **1c** (**B**), **5** (**C**), **8** (**D**,**F**) and **7** (**E**,**G**) for 24 h (light gray) and 48 h (dark gray). The values are expressed as percentages of alive cells ± SD. ^#^ *p* < 0.01; * *p* < 0.001.

All four tumor cell lines showed responsiveness; however, the FaDu cell line was the most responsive to all four compounds, as evidenced in a histogram (Figure 3).

The HaCaT cell line was treated with **7** and **8**, the two compounds with the greatest activity as inhibitors of proliferation in cancer cells, and there was no significant effect except at a very high concentration, above 100 µM (Figure 3).

## 3. Materials and Methods

### 3.1. Chemistry

The synthesis of pyrrolyl and indolyl DKA derivatives **1a–d, 2a–d, 3a,b, 4a,b** and **5–8** are reported in the literature [49,50,51].

### 3.2. Molecular Modelling

The crystal structures of hCA II (pdb 5LJT) and hCA IX (pdb 5FL4) used in the computational studies were prepared using the Protein Preparation Wizard tool implemented in the Schrödinger suite [52], assigning bond orders, adding hydrogens, deleting water molecules and optimizing H-bonding networks. The energy minimization protocol with a Root Mean Square Deviation (RMSD) value of 0.30 Å was applied using an Optimized Potentials for Liquid Simulation (OPLS4) force field. The 3D ligand structures were prepared by Maestro (v.12.9) and evaluated for their ionization states at pH 7.4 with Epik (v.5.7). The conjugate gradient method in Macromodel (v.13.3) was used for energy minimization (maximum iteration number: 2500; convergence criterion: 0.05 kcal mol*^−^*^1^Å*^−^*^1^). The software Glide SP (v.9.2) was used for molecular docking studies. Grids were centered in the centroid of the complexed ligand. The standard precision (SP) mode of the Glide Score function was applied to evaluate the predicted binding poses. The best-scored poses were refined with Prime MM-GBSA calculations (v.5.5) with a VSGB (Variable Surface Generalized Born) solvation model considering the target flexible within 3 Å around the ligand. Figures were generated with Chimera.

### 3.3. Carbonic Anhydrase Inhibition

An Applied Photophysics stopped-flow instrument has been used for assaying the CA catalyzed CO_2_ hydration activity [48]. Phenol red (at a concentration of 0.2 mM) has been used as an indicator, working at the absorbance maximum of 557 nm, with 20 mM Hepes (pH 7.5) as buffer and 20 mM Na_2_SO_4_ (for maintaining constant the ionic strength), following the initial rates of the CA-catalyzed CO_2_ hydration reaction for a period of 10−100 s. The CO_2_ concentrations ranged from 1.7 to 17 mM for the determination of the kinetic parameters and inhibition constants. For each inhibitor, at least six traces of the initial 5−10% of the reaction have been used for determining the initial velocity. The uncatalyzed rates were determined in the same manner and subtracted from the total observed rates. Stock solutions of inhibitor (0.1 mM) were prepared in distilled−deionized water and dilutions up to 0.01 nM were performed thereafter with the assay buffer. Inhibitor and enzyme solutions were preincubated together for 15 min at room temperature before assay to allow for the formation of the E−I complex. The inhibition constants were obtained by nonlinear least-squares methods using PRISM 3 and the Cheng−Prusoff equation, as reported earlier [53], and they represent the mean from at least three different determinations. The enzyme concentrations were in the range 3–11 nM. All hCA isoforms were recombinant ones obtained in-house as reported earlier [54].

### 3.4. Biological Assays

#### 3.4.1. Cell Cultures and Treatments

The following human tumor cell lines were utilized in the present study: triple-negative breast cancer MDA-MB231, colon carcinoma HT-29, tongue squamous cell carcinoma SCC-15 and pharynx squamous cell carcinoma FaDu. In addition, a spontaneously transformed keratinocyte cell line from human skin HaCaT was used as control of non-transformed cells. All cell lines were purchased from the American Type Culture Collection (Manassas, VA, USA).

The cell lines were grown in RPMI 1640 medium supplemented with 10% fetal calf serum (FCS), 2 mM glutamine and 50 U/mL penicillin–streptomycin.

All compounds were solubilized in dimethylsulfoxide (DMSO) (Sigma) for a 10 mM stock solution and utilized to final concentrations from 5 to 100 μM for 24 and 48 h. Control cells were treated with equivalent amounts of DMSO in every experiment.

#### 3.4.2. Cytotoxicity Assay

To determine cytotoxicity, a sulforhodamine B colorimetric assay was performed: 5 × 10^3^ cells were plated on 96-well plates, grown for 24 h and treated with different concentrations of each compound for 24 and 48 h. Cells were then fixed with 50% trichloroacetic acid for 1 h at 4 °C and stained for 30 min (min) at room temperature (RT) with 0.4% sulforhodamine B in 1% acetic acid. Excess dye was removed by washing four times with 1% acetic acid. Protein-bound dye was dissolved in 10 mM Tris pH 10 and optical density was determined at 510 nm using a microplate reader.

#### 3.4.3. Statistical Analysis

All results were analyzed using one-way analysis of variance, and significance was evaluated using Tukey’s honest significant difference post hoc test. All experiments were repeated at least three times. The statistical analyses and the graphs were conducted using Graph Pad Prism 5.0.

## 4. Conclusions

Herein, we reported a new set of diketo acid derivatives as potent and selective CAIs active towards the CA isoforms IX and XII, highly expressed in tumor cells, and scarcely or not active against the cytosolic CAs I and II. In general, the acid derivatives reported higher inhibitory potencies than the corresponding ester counterparts against all the tested isoforms. However, we found that the ester derivative **8**, despite having lower enzymatic inhibitory potencies than its acid counterpart **7**, showed a comparable efficacy in reducing tumor cell proliferation. We speculated that this effect could be ascribed to the higher permeability of the ester form across cell membranes, leading to a significant increase in cellular uptake. As a consequence, the acid and ester forms, tested at the same concentrations, determined comparable effects on treated cells, despite their different enzymatic inhibitory potencies.

The best acting compounds against both CA IX and XII were the acids **1c**, **3a**, **5** and **7**, with the most potent compounds **1c** and **5** as nanomolar inhibitors of CA IX and XII, being also two orders of magnitude more selective against CA IX and XII vs. I and II. Molecular modeling studies performed on the best acting compounds **1c**, **3a**, **5** and **7** within CA II and IX active sites showed the different binding poses of the best acting CAIs within CA II and IX, highlighting the coordination of the zinc ion by the carboxylate moiety of DKAs with a tetrahedral geometry. In comparison with the binding mode within the CA II active site, the better accommodation of the ligands into the CA IX binding site accounted for their higher selectivity toward CA IX. Furthermore, all the tested compounds showed a dose-dependent reduction in viability already after 24 h treatment in different tumor cell lines overexpressing CA IX and XII, except for **1b**, that showed only a low efficacy in inhibiting the proliferation, exclusively at higher concentrations and times of treatment. Noteworthy, derivatives **7** and **8**, despite being endowed with different inhibitory activities against hCA IX and hCA XII, proved to be the compounds with the greatest activity as inhibitors of proliferation in cancer cells. They also showed no significant effect on somatic not transformed cells, except at very high concentration. Taken together, these data suggest a potential role for this class of molecules as a promising tool for new approaches in treating different solid cancer types.

## Figures and Tables

**Table 1:** Inhibition data of hCA isoforms I, II, IX and XII using acetazolamide (AAZ) as standard drug.

Cpd	R	R_1_	X	K*_i_* (µM) ^1^			
				CA I	CA II	CA IX	CA XII
**1a**	H	4-FBn ^2^	H	81.9 ± 6.0	14.6 ± 0.9	2.0 ± 0.1	1.0 ± 0.1
**2a**	H	4-FBn	Et	>100	>100	90.1 ± 4.7	83.6 ± 5.4
**1b**	H	2-FBn	H	89.6 ± 4.8	14.0 ± 0.8	2.5 ± 0.1	2.2 ± 0.1
**2b**	H	2-FBn	Et	>100	>100	23.7 ± 1.6	80.5 ± 6.1
**1c**	H	4-CH_3_Bn	H	79.0 ± 3.9	44.4 ± 2.6	0.82 ± 0.05	0.90 ± 0.07
**2c**	H	4-CH_3_Bn	Et	>100	>100	90.8 ± 4.6	89.3 ± 5.3
**1d**	Bz ^3^	-	H	72.5 ± 5.1	38.7 ± 2.0	1.4 ± 0.1	1.1 ± 0.1
**2d**	Bz	-	Et	nd ^5^	nd	nd	nd
**3a**	Ph ^4^	-	H	>100	77.6 ± 4.5	1.4 ± 0.1	0.33 ± 0.02
**4a**	Ph	-	Et	>100	>100	57.3 ± 2.8	89.2 ± 5.2
**3b**	H	-	H	50.9 ± 4.1	54.7 ± 2.9	18.4 ± 1.2	7.9 ± 0.6
**4b**	H	-	Et	>100	>100	>100	71.5 ± 4.3
**5**	-	-	H	>100	62.5 ± 3.6	0.32 ± 0.02	0.24 ± 0.02
**6**	-	-	Et	>100	>100	43.1 ± 2.5	89.0 ± 6.1
**7**	-	-	H	91.7 ± 7.5	36.5 ± 1.9	1.1 ± 0.1	0.83 ± 0.06
**8**	-	-	Et	>100	>100	80.1 ± 5.3	92.0 ± 6.0
**AAZ**	-	-		0.250	0.012	0.025	0.0057

^1^ Inhibition constant (μM) by a stopped flow technique expressed as means ± SEM of 3 different assays. ^2^ Bn = benzyl. ^3^ Bz = benzoyl. ^4^ Ph = phenyl. ^5^ not determined.

**Table 2:** Antiproliferative activities of compounds **1b,c, 5, 7** and **8**.

Cpd	EC_50_ (µM) ^1^			
	MDA-MB231	HT-29	FaDu	SCC-15
**1b**	nd ^2^	92 ± 9.9 (24 h)	98 ± 8.6(24 h)	nd
	nd	75 ± 8.2 (48 h)	90 ± 9.3(48 h)	nd
**1c**	85 ± 9.1 (24 h)	115 ± 10.8 (24 h)	59 ± 4.5 (24 h)	96 ± 8.5 (24 h)
	73 ± 5.5 (48 h)	48 ± 4.1 (48 h)	17 ± 0.9 (48 h)	53 ± 5.9 (48 h)
**5**	120 ± 15.7 (24 h)	98 ± 11.0 (24 h)	103 ± 11.4 (24 h)	102 ± 9.7 (24 h)
	105 ± 13.2 (48 h)	21 ± 1.6 (48 h)	23 ± 2.8 (48 h)	58 ± 6.2 (48 h)
**7**	21 ± 1.8 (24 h)	47 ± 5.0 (24 h)	51 ± 4.7 (24 h)	48 ± 3.9 (24 h)
	12 ± 1.1 (48 h)	30 ± 2.8 (48 h)	22 ± 1.2 (48 h)	17 ± 0.8 (48 h)
**8**	23 ± 1.6 (24 h)	75 ± 8.8(24 h)	41 ± 3.3(24 h)	44 ± 4.1 (24 h)
	17 ± 0.9 (48 h)	44 ± 2.3 (48 h)	18 ± 0.7(48 h)	21 ± 1.9 (48 h)

^1^ Half maximal effective concentration (μM) on five different cell lines at 24 and 48 h ± SD. ^2^ not determined.

## Data Availability

Data are contained within the article.

## Sample Availability

Samples of the compounds **1a–d, 2a–d, 3a,b, 4a,b** and **5–8** are available from the authors.
